# Attitudes and Stigma Toward Seeking Psychological Help Among the General Population of Makkah, Saudi Arabia

**DOI:** 10.7759/cureus.55492

**Published:** 2024-03-04

**Authors:** Mohammed A Aljuhnie, Abdullah S Alharbi, Omar F Alharbi, Asim A Saati, Fahad A Alshumrani, Abdullah E Alharbi, Raghad F Hazazi, Mohammad S Alharbi, Mokhtar Shatla

**Affiliations:** 1 Department of Medicine and Surgery, College of Medicine, Umm Al-Qura University, Makkah, SAU; 2 Department of Physical Therapy, College of Applied Medical Sciences, Umm Al-Qura University, Makkah, SAU; 3 Infectious Disease Control Department, Saudi Ministry of Health, Makkah, SAU; 4 Department of Community Medicine and Pilgrims Health Care, College of Medicine, Umm Al-Qura University, Makkah, SAU

**Keywords:** saudi arabia, makkah region, mental health stigma, help-seeking attitudes, seeking psychological help

## Abstract

Background and objective

Depression and anxiety are among the most common mental health conditions globally, and, according to the World Health Organization (WHO), roughly 25% of people worldwide suffer from them. Serious mental diseases can cause a great deal of suffering and incapacity, lowering people’s quality of life. Stigma and unfavorable attitudes toward mental illness often discourage people from seeking psychological assistance and achieving recovery from mental problems. This observational cross-sectional study aimed to investigate the attitudes of the general population of Makkah, Saudi Arabia, toward seeking psychological help, and to determine the degree to which stigma prevents individuals from seeking help.

Methods

An online, self-administered survey was distributed via social media platforms among the general population of Makkah between September and December 2023. Males and females over the age of 18 years living in Makkah were included. The exclusion criteria were participants who declined to participate in the study or those who were below 18 years of age.

Results

A total of 495 eligible participants completed the study survey. Of them, 378 (76.4%) were female, and most (390, 78.8%) were Saudi Arabian nationals. A total of 341 (68.9%) participants had symptoms of anxiety, and 319 (64.4%) had symptoms of depression. Regarding unfavorable attitudes, the scores were significantly higher among participants over 40 years of age (1.81 ± 0.46; p<0.05) and those with relatively low levels of education (1.93 ± 0.65; p<0.05). As for stigma, the scores were significantly higher among male participants (2.38 ± 0.83; p<0.05) and those with low levels of education (2.54 ± 0.8; p<0.05).

Conclusion

A significant negative correlation between participants’ attitudes toward seeking psychological help and stigma was observed. However, in contrast, the psychological symptom scores did not significantly correlate with the participants’ attitudes. Stigma scores showed significant positive correlations with depression and overall symptom scores. This research showed that stigma has a significant impact on attitudes toward help-seeking.

## Introduction

Depression and anxiety rank among most the prevalent medical issues globally [1]. According to the World Health Organization (WHO), they affect roughly 25% of people worldwide [2]. Serious mental diseases can cause a great deal of suffering and incapacity, significantly affecting people’s quality of life [3]. Major mental disorders may have an acute onset, with people quickly exhibiting full-blown sickness, or an insidious beginning, with the illness gradually manifesting over time [3]. Stigma and unfavorable attitudes toward mental illness often discourage people from seeking psychological assistance and achieving recovery from mental problems [4].

Mental health stigma

Erving Goffman, who first identified the aspect of stigma in 1963, defined it as any trait or quality that causes someone to be undervalued, tarnished, or regarded with shame or dishonor [5]. Cultural and contextual value systems that vary over time and between contexts have a significant impact on stigma [5]. Mental health stigma is defined as shame, social rejection, and/or social discrediting experienced by people with mental health issues [5]. Multiple dimensions or types of mental health stigma have been identified in the literature, including self-stigma, public stigma, professional stigma, and institutional stigma [5].

Stigma related to psychological help-seeking

The complex nature of stigma may discourage people from obtaining psychiatric assistance [6]. A Finnish study has explored the theory of sociopsychological two-factor stigma, distinguishing between self-stigmatization and public stigmatization [7]. Self-stigmatization refers to an individual perceiving him/herself as a member of a stigmatized group. In contrast, public stigmatization refers to how the general public reacts to a particular group of stigmatized individuals [7]. Researchers have discovered that help-seeking attitudes are potent indicators of intentions to seek psychological care [8-10]. Although there have been huge advances in the understanding and recognition of help-seeking behavior, widespread unfavorable views persist in Saudi Arabia about people with mental illnesses [11]. Even today, a sizable segment of the population still opt not to receive psychological care despite experiencing psychological distress [11]. A literature review revealed one study similar to ours conducted in the Eastern Province of Saudi Arabia [11]. Our study is the first of its kind to be conducted in Makkah, Saudi Arabia, and we aimed to investigate Saudi Arabian adults’ attitudes toward seeking psychological help, assess levels of stigma, and determine the degree to which stigma prevents individuals from seeking psychological help.

## Materials and methods

Study design and participants

We employed convenience sampling to recruit participants for this study. The inclusion criteria were males and females over the age of 18 years living in Makkah. The exclusion criteria were participants who declined to participate in the study or those who were below 18 years of age.

Ethical considerations and sample size

We used social media platforms to distribute an online, self-administered survey, which included a consent form at the beginning of the questionnaire to be signed by the participant before starting, to the general population of Makkah between September and December 2023. Umm Al-Qura University’s Biomedical Ethics Committee gave ethical approval for the study (approval no. HAPO-02-K-012-2023-09-1746). We then employed OpenEpi software (version 3.01) to determine a minimum sample size of 385 participants, with a 95% confidence interval and a 5% p-value [12]. To improve the generalizability and precision of the results, we enrolled 495 participants in the study. There was no missing data to be handled.

Study tool

The first section of the survey included sociodemographic data, including age, gender, marital status, occupation, education level, and socioeconomic status. The items in the second section were based on the Arabic edition of the Attitudes Toward Seeking Professional Psychological Help Scale-Short Form (ATSPPH-SF-A), which measures participants’ attitudes toward seeking professional assistance for psychological issues using a 10-item scale [13,14]. A 4-point Likert-type scale (ranging from 0 = disagree to 3 = agree) was employed to rate the items. Thereafter, we summed up all the item scores, with higher scores denoting positive attitudes toward psychological help. Half of the items (2, 4, 8, 9, and 10) were reversed, yielding an overall ATSPPH-SF-A score. Following validation with a select group of university students from the United Arab Emirates, the instrument’s (low) Cronbach’s alpha value was 0.65, close to the 0.67 reported by Vogel et al. [15].

The third section of the survey consisted of the Arabic version of the Stigma Scale for Receiving Psychological Help (SSRPH-A) [13,16], which is a self-reported, five-item test designed to assess the stigma associated with seeking psychological help. Each item was rated using a 4-point Likert scale (ranging from 1 = strongly disagree to 4 = strongly agree) [16]. We summed up the scores for the five items to obtain the SSRPH-A overall score, with higher scores corresponding to greater degrees of stigma. A group of university students from the United Arab Emirates helped us validate the tool, and a reported alpha value of 0.70 was deemed acceptable [13]. The Arabic version of the Hopkins Symptom Checklist-25 (HSCL-25-A), which was validated in a study conducted in Lebanon [17], comprised the final section of the survey. The 25-question HSCL-A self-reported questionnaire is divided into two sections: the first part contains 10 items related to anxiety symptoms, and the second part consists of 15 items related to depression symptoms. A 4-point response scale (ranging from 1 = not at all to 4 = extremely) was provided for each question. We determined the scores based on symptoms reported during the previous week. To calculate the scores for depression and anxiety symptoms, we summed up the response scores and divided the total by the total number of items answered. The cutoff point for scientific validity was 1.75; participants with scores higher than this value were deemed to have symptoms of anxiety and/or depression. The Cronbach’s alpha value was 0.90.

Statistical analysis

The data were collected, reviewed, and then input to the IBM® SPSS® Statistics version 21 (IBM Corp., Armonk, NY). We used two-tailed statistical methods, with an alpha level of 0.05, and a p-value ≤0.05 was considered significant. We conducted descriptive analysis by prescribing frequency distributions and percentages for the study variables and the participants’ sociodemographic data, including age, gender, education, and economic status. Mean scores were presented for all scales with standard deviations (SD). We conducted a Pearson’s chi-squared test for significance and an exact probability test for small frequency distributions. We used a one-way ANOVA to compare the scores for stigma, psychological distress, and attitudes toward psychological help-seeking for independent variables with more than two categories, such as age group, education level, and economic status. We performed an independent t-test for the statistical testing of variables with only two categories (e.g., gender and nationality). We employed Pearson’s correlation analysis to assess the correlations among stigma, psychological distress, attitudes toward psychological help-seeking, and symptoms of anxiety and depression. For the factors associated with people’s attitudes toward seeking psychological help, we included all statistically significant univariate correlations in the multiple linear regression.

## Results

A total of 495 eligible participants completed the study survey. The participants’ ages ranged from 18 to 76 years, with a mean age of 28.6 ± 11.5 years. Of them, 378 (76.4%) were female, and most (390, 78.8%) were Saudi Arabians. Regarding education level, 337 (68.1%) had a university education, 100 (20.2%) had a secondary-level education, and 34 (6.9%) had a postgraduate degree. A total of 322 (65.1%) study participants had an average economic status, but 142 (28.7%) reported an excellent economic status (Table 1).

**Table 1:** Socioeconomic data of study participants

Variable	Number	Percentage
Age in years		
<20	73	14.7%
20-25	195	39.4%
25-30	67	13.5%
31-40	79	16.0%
>40	81	16.4%
Gender		
Male	117	23.6%
Female	378	76.4%
Nationality		
Saudi	390	78.8%
Non-Saudi	105	21.2%
Educational level		
Below secondary	24	4.8%
Secondary	100	20.2%
University/above	337	68.1%
Postgraduate	34	6.9%
Economic status		
Below average	31	6.3%
Within average	322	65.1%
Excellent	142	28.7%

Table 2 shows the anxiety and depression symptoms among the study participants. A total of 341 (68.9%) participants had symptoms of anxiety, and 319 (64.4%) had symptoms of depression.

**Table 2:** Anxiety and depression symptoms among study participants

Hopkins Symptom Checklist	Number	Percentage
Anxiety		
Asymptomatic	154	31.1%
Symptomatic	341	68.9%
Depression		
Asymptomatic	176	35.6%
Symptomatic	319	64.4%

Table 3 presents a comparison of the mean scores for attitudes toward seeking psychological help, stigma, and psychological distress relative to the participants’ sociodemographic and clinical characteristics. Regarding attitudes, the scores were significantly higher among participants over 40 years of age (1.81 ± 0.46; p<0.05) and those with relatively low levels of education (1.93 ± 0.65; p<0.05). As for stigma, the scores were significantly higher among male participants (2.38 ± 0.83; p<0.05) and those with low levels of education (2.54 ± 0.8; p<0.05). The psychological symptom scores were significantly higher among young participants aged 20-25 years (2.21 ± 0.59; p<0.05) and among females (2.13 ± 0.6; p<0.05). Regarding attitudes toward seeking psychological help, the participants’ mean scores were 1.70 ± 0.46 out of 3. In addition, the mean scores for stigma were 2.21 ± 0.73 out of 4. The mean scores for Hopkins symptoms were 2.07 ± 0.61 out of 4.

**Table 3:** Comparison of attitudes toward seeking psychological help, stigma, and psychological distress based on sociodemographic and clinical characteristics of participants SD: standard deviation; ATSPPH-SF: Attitudes Toward Seeking Professional Psychological Help Scale-Short Form; HSL-25: Hopkins Symptom Checklist-25

Sociodemographics	ATSPPH-SF		Stigma score		HSL-25 score	
	Mean	SD	Mean	SD	Mean	SD
Age in years						
<20	1.69	0.41	2.15	0.69	2.15	0.65
20-25	1.63	0.44	2.14	0.71	2.21	0.59
25-30	1.69	0.55	2.38	0.67	2.02	0.57
31-40	1.75	0.43	2.19	0.77	1.96	0.66
>40	1.81	0.46	2.31	0.77	1.81	0.49
P-value	0.026		0.111		0.001	
Gender						
Male	1.75	0.47	2.38	0.83	1.88	0.58
Female	1.68	0.45	2.16	0.68	2.13	0.60
P-value	0.162		0.005		0.001	
Nationality						
Saudi	1.69	0.47	2.21	0.75	2.06	0.62
Non-Saudi	1.73	0.42	2.21	0.63	2.12	0.56
P-value	0.414		0.937		0.364	
Educational level						
Below secondary	1.93	0.65	2.54	0.80	2.12	0.70
Secondary	1.74	0.42	2.20	0.75	2.08	0.61
University/above	1.67	0.45	2.17	0.70	2.09	0.61
Postgraduate	1.71	0.45	2.40	0.83	1.85	0.54
P-value	0.043		0.043		0.194	
Economic status						
Below average	1.75	0.56	2.32	0.76	2.22	0.64
Within average	1.71	0.42	2.17	0.68	2.04	0.58
Excellent	1.65	0.51	2.28	0.80	2.10	0.66
P-value	0.374		0.216		0.232	
Overall Mean ± SD	1.7 ± 0.45		2.2 ± 0.73		2.1 ± 0.61	

Table 4 shows the correlations between the participants’ attitudes, psychological symptoms, and stigma regarding seeking psychological help. There was a significant negative correlation between participants’ attitudes toward seeking psychological help and stigma (r = 0-.204; p = 0.004), but the psychological symptom scores did not significantly correlate with the participants’ attitudes. Stigma scores showed significant positive correlations with depression and overall symptom scores (r = 0.12 and 0.089, respectively), but not with anxiety.

**Table 4:** Correlation between participants' attitudes, psychological symptoms, and their stigma toward seeking psychological help ATSPPH-SF: Attitudes Toward Seeking Professional Psychological Help Scale-Short Form; HSL-25: Hopkins Symptom Checklist-25

Scales	ATSPPH-SF score	Stigma score	Anxiety score	Depression score	HSL-25 score
ATSPPH-SF	1	0.204	0.029	0.057	0.048
Stigma score	-	1	0.034	0.120	0.089
Anxiety score	-	-	1	0.756	-
Depression score	-	-	-	1	-
HSL-25 score	-	-	-	-	1

Table 5 shows the multiple stepwise linear regression results for the factors associated with the participants’ attitudes toward seeking psychological help. Among the included factors, stigma, participants’ age, and education level were the most significant determinants of their attitudes. Greater stigma and higher levels of education were associated with negative attitudes (ß = -0.12 and -0.06, respectively), but higher education was associated with positive attitudes (B = 0.04).

**Table 5:** Multiple stepwise linear regression for assessing factors associated with participants' attitudes toward seeking psychological help B: regression coefficient; SE: standard error; t: statistical test for p-value; R^2^: coefficient of determination; AdjR^2^: adjusted regression coefficient

Factors	Unstandardized coefficients		Standardized coefficients	t	P-value	R^2^	AdjR^2^
	B	SE	Beta				
(Constant)	1.48	0.12				0.11	0.09
Stigma score	-0.12	0.03	0.19	4.36	0.001		
Age	0.04	0.01	0.11	2.50	0.013		
Educational level	-0.06	0.03	-0.09	-2.04	0.042		

## Discussion

The results indicated that attitudes toward seeking professional help for mental health issues in Makkah, Saudi Arabia, depend on the cultural context and can shape individuals’ inclinations toward seeking psychological assistance. These inclinations can be influenced by various elements, such as educational background, familial principles, cultural perspectives on mental well-being and disorders, and the availability of mental healthcare facilities. In Arab culture, there is still a prevalent perception that seeking psychological assistance indicates weakness or mental instability [17]. People in Makkah tend to rely more on traditional methods, such as seeking support from family, friends, and religious figures, and/or finding solace in the Holy Qu’ran, when dealing with psychological challenges [17].

A total of 495 people completed the questionnaire for this study. The participants’ ages ranged from 18-76 years, and 76.4% of the participants were female. Most of the participants (78.8%) were Saudi Arabians. In terms of education level, 68.1% had a university education, 20.2% had a secondary level of education, and 6.9% had a postgraduate degree. Regarding economic status, 65.1% of the participants had an average economic status, whereas 28.7% reported an excellent economic status. Of note, 68.9% of the participants had symptoms of anxiety, and 64.4% had symptoms of depression. We observed positive attitudes toward psychological issues among participants with lower levels of education and older participants. This finding contrasts with that of a previous study conducted in Singapore [13], which was based on a survey of Singapore residents aged 18-65 years. This difference could be attributed to the wider age range among the sample in the Singaporean study, since the perspectives and experiences of older individuals may differ from those of younger people. Additionally, the survey distribution and sampling techniques captured a more diversely representative sample of the population, leading to different results compared to previous research that focused on specific subgroups, which showed a significant correlation between a younger age group (18-34 years) and a greater inclination to be open to seeking professional psychological assistance [13].

Our findings showed that the stigma scores were significantly higher among male participants and those with lower levels of education, consistent with previous research conducted on mental stigma among the general Saudi Arabian population [18]. Additionally, a study on the facilitators of and barriers to seeking mental health help in Saudi Arabia found frequent delays in seeking support for psychological well-being due to barriers to seeking help, such as public stigma, difficulty in accessing resources and information, lack of qualified mental health professionals, insufficient knowledge, and unsupportive family members [19]. Another systematic review conducted in the Caribbean indicated that mental health stigma was negatively associated with help-seeking and suggested that stigma can lead to the underutilization of mental health services [20].

A study conducted in Abha, Saudi Arabia, reported a high prevalence of mental illness symptoms among secondary school girls, with phobic anxiety being the most prevalent symptom (16.4% of participants), followed by psychoticism, anxiety, and somatization [21]. This finding suggests that mental health issues, particularly anxiety-related symptoms, are prevalent among young females in Saudi Arabia [21]. Furthermore, a meta-analysis and systematic review conducted on the general population of Saudi Arabia during the COVID-19 outbreak revealed high levels of stress, anxiety, and depression [22]. The study found that 36.5% of the population experienced mild to extremely severe levels of stress, 34.9% experienced anxiety, and 43.5% experienced depression. These findings indicate the significant impact of the pandemic on mental health in Saudi Arabia [22]. The results of the current research in Makkah, Saudi Arabia, which revealed higher psychological symptom scores among participants aged 20-25 years and females and showed a significant negative correlation between participants’ views regarding obtaining mental health help and stigma, align with previous studies suggesting the vulnerability of these groups to mental health issues [21,22].

The psychological symptom scores in the current study showed insignificant correlations with the participants’ attitudes. However, the stigma scores showed significant positive correlations with depression and overall symptom scores but not with anxiety symptom scores. This suggests that stigma may be more strongly associated with depression and overall psychological symptoms than with anxiety. The findings highlight the importance of addressing stigma in the context of mental health, particularly concerning depression and overall psychological symptoms [23,24]. Another study regarding the relationships between stigma, depression, and anxiety in a Saudi Arabian population recovering from COVID-19 found that the psychological symptom scores significantly correlated with participants’ attitudes, while the stigma scores showed significant negative correlations with depression and overall symptom scores, but not with anxiety [25].

The results revealed that stigma, age, and education level were the most significant determinants of the participants’ attitudes, aligning with a study conducted in 2020 to assess primary care patients’ attitudes toward asking for help [26]. This study showed that education, attitudes toward seeking help, and personal stigma were inversely correlated, such that more negative attitudes toward seeking help were linked to higher education levels and higher levels of personal stigma, depending on a combination of socioeconomic characteristics, including elements related to behavioral health and education. Furthermore, the results showed that high stigma and low education levels were associated with negative attitudes, while higher education levels were associated with positive attitudes.

Despite its significant findings, this study has several limitations. Our sample may not have been entirely representative of Makkah’s general population, thus influencing how broadly the findings can be applied. The attitudes of Saudi Arabian people may differ from those of other cultures regarding seeking professional psychological help. In addition, the questionnaire did not address prior contact with mental health services. Moreover, the participants who intended to seek help may have responded in a socially desirable manner, resulting in bias. We recommend further studies on this topic, with different study designs, sampling techniques, and methods of data collection. Nevertheless, this study has several notable strengths. Firstly, it offers insights into an under-researched field by investigating public opinions about seeking psychological support in the specific context of Makkah, Saudi Arabia. It fills a crucial research gap and adds to the body of knowledge as the first study of this type in the Makkah area. Second, the study had a comparatively large sample of 495 participants, which improved the statistical power and reliability of the results. Thanks to the wide range of age groups, educational backgrounds, and professions represented by the participants, the study provides a thorough understanding of the public’s attitudes about seeking psychological assistance.

## Conclusions

The results of the current study showed significant negative correlations between participants’ attitudes toward seeking psychological help and stigma. However, in contrast, the psychological symptom scores did not significantly correlate with the participants’ attitudes. Stigma scores showed significant positive correlations with depression and overall symptom scores. We believe our findings contribute to the literature showing that stigma leads to the inadequate utilization of resources due to the reluctance among people who need psychological assistance to seek help. Additionally, this research showed that stigma has a significant impact on help-seeking attitudes. Gaining a deeper understanding of the sociocultural aspects of mental health stigma in Saudi Arabia and how it affects attitudes toward seeking help is undoubtedly necessary.
